# VCP/p97, Down-Regulated by microRNA-129-5p, Could Regulate the Progression of Hepatocellular Carcinoma

**DOI:** 10.1371/journal.pone.0035800

**Published:** 2012-04-20

**Authors:** Yu Liu, Yan Hei, Qingming Shu, Jie Dong, Yaping Gao, Hanjiang Fu, Xiaofei Zheng, Guang Yang

**Affiliations:** 1 Beijing Institute of Basic Medical Sciences, Beijing, China; 2 People's Armed Police Corps General Hospital, Beijing, China; 3 Beijing Institute of Radiation Medicine, Beijing, China; University of Hong Kong, Hong Kong

## Abstract

Valosin containing protein (VCP)/p97 plays various important roles in cells. Moreover, elevated expression of VCP in hepatocellular carcinoma (HCC) is correlated with increased incidence of recurrence. But the role of VCP in HCC progression *in vitro* and *in vivo* is unclear. And there are few reports about the regulation mechanism on the expression of VCP in HCC. In this study, it was identified that the level of VCP was frequently increased in human HCC tissues. In addition, down-regulation of VCP with siRNAs could dramatically suppress the genesis and progression of tumor *in vivo*. It was found that miR-129-5p directly inhibited the expression of VCP in several HCC cell lines. Meanwhile, the level of VCP in HCC tissues was negatively associated with the level of miR-129-5p. Our further investigation showed that the enhanced expression of miR-129-5p also suppressed tumor growth *in vivo*. Moreover, it was revealed that miR-129-5p could inhibit the degradation of IκBα and increase the apoptosis and reduce the migration of HCC cells by suppressing the expression of VCP. Our results revealed that the expression of VCP was directly regulated by miR-129-5p and this regulation played an important role in the progression of HCC.

## Introduction

Hepatocellular carcinoma (HCC) is one of the common cancers worldwide, especially in Southeast Asia and Africa [1]. Surgical resection is the main modality of treatment for HCC, but the recurrence rate of HCC after surgery is about 70%. The results of immunohistochemistry show that elevated expression of Valosin-containing protein (VCP), also named p97, is correlated with increased incidence of HCC recurrence [2]. But there is no direct evidence that VCP is involved in the progression of HCC.

VCP belongs to the AAA family (ATPase with multiple cellular activities) [3]. It is one of the most abundant proteins in eukaryotic cells and can interact with more than 30 different cellular proteins with various functions, including morphology alteration of nuclear and Golgi membranes, transcriptional regulation, membrane fusion, programmed cell death, ubiqutin/protesome-dependent protein degradation, ER-associated degradation [3]–[7]. In addition, VCP has been shown to associate physically with ubiquitinated IκBα and to target IκBα to the proteasome for degradation [7], [8]. It was reported that pre-B-cell leukemia transcription factor 1(PBX1) regulates expression of VCP [9]. But so far there is no report about the post-transcriptional regulation of the VCP expression.

microRNAs (miRNAs) are a class of small non-coding RNAs in animals and plants. They can bind to the 3′ untranslated regions (UTRs) of target mRNAs and regulate genes expression at posttranscriptional and translational levels [10]. Deregulation of miRNAs is involved in many kinds of human diseases, including cancer [11]. It has been shown that 50% of miRNAs are located within the chromosomal regions known to be frequently amplified or deleted in human cancer cells [12]. Until now, several miRNAs are reported to be associated with HCC, such as miR-21, miR-221, miR-223, miR-224, miR-122, miR-199a/b-3p, miR-101, miR-26 and miR-29 [12]–[20].

Here, we firstly identified miR-129-5p could directly regulate the expression of VCP and inhibit the degradation of IκBα. Suppression of the expression of VCP or increasing the level of miR-129-5p could induce cell apoptosis and migration *in vitro* and inhibit the tumor genesis of HCC *in vivo*.

## Methods

### Ethics Statement

All animal experimental protocols of the study are in accordance with the national guidelines for the use of animals in scientific research. It's also approved by Animal Care and Use Committee of Beijing Institute of Basic Medical Sciences, with the approval number BMS-1104139.

### Patients Characteristics

The liver tissue specimens were obtained at People's Armed Police Corps General Hospital (Beijing, China). The institutional ethics committee of People's Armed Police Corps General Hospital approved the study, and all patients gave written informed consent. All specimens were clinically characterized (Tables S1 and S2).

### Cell Lines

The HepG2 [17] and SK-HEP1 [21] cell lines were purchased from the Cell Bank of Type Culture Collection of Chinese Academy of Sciences (Shanghai, China). MHCC-LM3 cell line [22] was provided by the Liver Cancer Institute of Fudan University (Shanghai, China). They were all maintained in Dulbecco's Modifed Eagle Medium (DMEM) (Gibco) supplemented with 10% fetal bovine serum (FBS) with 100 U/ml penicillin and 100 U/ml streptomycin. Cells were cultured at 37°C in 5% CO_2_.

### RNA extraction and cDNA production

Total RNA was extracted from cells or tissues using Trizol (Invitrogen) according to the manufacturer's protocol. microRNAs were extracted from paraffin-embedded sections by using the RecoverAll™ Total Nucleic Acid Isolation Kit (Applied Biosystems). For cDNA synthesis,1 µg of RNA was mixed with 500 ng of olig (dT) (Promega) or microRNA specific primers (invitrogen). The RNA-primer mixture was incubated at 70°C for 10 min, followed by a snap freeze in ice bath for 2 min. Then samples were incubated at 42°C for 45 min with 5 µl of 5×first-strand buffer, 2 µl of 5 mM dNTP, 20 U of RNasin (Takara), 1 µl of M-MLV reverse transcriptase (Promega) and distilled water to a total volume of 25 µl. The reverse transcriptase was inactivated at 70°C for 10 min and then chilled on ice.

### Quantitative reverse transcription PCR (qRT-PCR)

The qPCR reaction mixture contained 12.5 µl of 2×SYBR green PCR mix (GenePharma), 0.3 µM of gene-specific forward and reverse primers, and 1 µl of template, made up to a final volume of 25 µl with distilled water. The primers are shown in Table S3. Cycling parameters were set as follows: initial activation step at 95°C for 10 min, denaturation at 95°C for 30 s, annealing at 58°C for 30 s, and extension at 72°C for 20 s. Melting curve analysis was performed at from 58°C to 95°C with stepwise fluorescence acquisition at every 1°C s^−1^. Melting curves observed for each gene were confirmed to correspond to the correct amplicon size by agarose gel electrophoresis of the PCR products. The levels of gene expression were calculated by relative quantification using GAPDH or U6 snRNA as the endogenous reference genes. All samples were amplified in triplicate and the data analysis was carried out using the MxPro qPCR system software (Stratagene).

### Vector construction and luciferase reporter assay

To generate a luciferase reporter construct, 3′UTR and mutant 3′UTR of VCP were inserted downstream of firefly luciferase in pGL3. Cells were cotransfected with miRNAs and 3′UTR or mutant 3′UTR luciferase reporters, using pRL-TK as control vector. Luciferase activity was measured using the Dual-Luciferase Assay kit (Promega) with a beta-counter luminometer. Relative luciferase activity was calculated as ratio of the raw firefly luciferase activity and the renilla luciferase activity.

### RNA oligoribonucleotides and cell transfections

Transfection of RNA oligoribonucleotide(s) was done using Lipofectamine 2000 (Invitrogen) according to the manufacturer's protocol. The transfection efficiency was examined by a FAM-conjugated siRNA (Genepharma). Sixty nM of RNA duplex or eighty nM of miRNA inhibitor were used for the transfection, unless otherwise indicated. In the rescue experiment, 24 h after RNA transfection, cells were transfected with 300 ng plasmids in a 24-well plate.

### Cell migration assay

HepG2 and SK-HEP1 cells were transfected and cultured for 24 h in DMEM containing 0.1% FBS. And then 1×10^5^ cells were harvested and added to upper chamber (8 µm pore size polycarbonate membrane, Corning) of 24-well plate in serum free medium (300 µl). After incubated for 24 h at 37°C in 5% CO_2_, invasive cells on lower surface of the membrane were stained with 0.1% violet staining solution for 30 min, and counted by photographing the membrane through the microscope (×40 magnifications).

### Tumor-bearing in nude mice

All experimental procedures involving animals were performed in accordance with the Guide for the Care and Use of Laboratory Animals National Institutes of Health and according to the institutional ethical guidelines for animal experiments. si-VCP, miR-129-5p- and NC-transfected HepG2 cells (1×10^6^) were suspended in 100 µl PBS and then injected subcutaneously into either side of the posterior flank of the male BALB/c athymic nude mouse at 5–6 weeks of age. 15 nude mice were included in each group and tumor growth was examined every four days. Tumor volume (V) was monitored by measuring the length (L) and width (W) of the tumor with calipers and was calculated with the formula V = (L×W^2^)×0.5.

### Statistical analysis

All quantitative data were analyzed using Student t-tests. All tests performed were two-sided. P<0.05 was considered to be statistically significant.

### Accession numbers

Human miR-129-5p, MIMAT0000242; homo sapiens VCP, NM_007126.3.

## Results

### Down-regulation of VCP suppressed the progression of HCC *in vivo*

**Figure 1:**
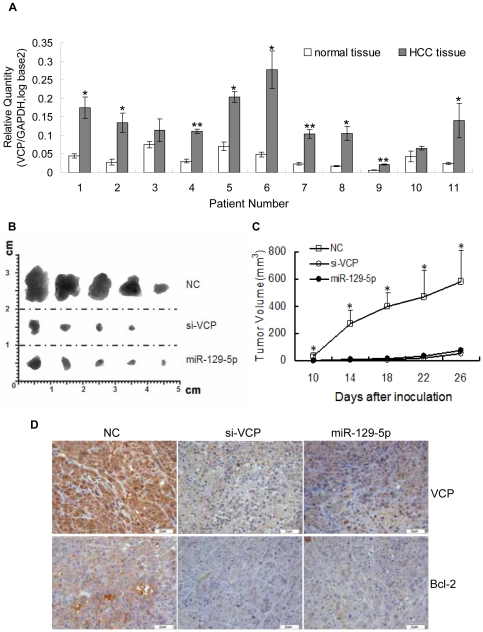
VCP positively regulated the progress of HCC. A: Analysis of the level of VCP in HCC tissues by qRT-PCR. The result showed that the level of VCP is frequently increased in human HCC tissues. *:P<0.05.**:P<0.01. B,C: Effect of si-VCP and miR-129-5p on tumor growth in nude mice model. si-VCP, miR-129-5p and NC-transfected HepG2 cells were suspended and then injected subcutaneously into either side of the posterior flank of the male BALB/c athymic nude mouse. Tumor growth was examined every four days. Tumor volume (V) was monitored by measuring the length (L) and width (W) of the tumor with calipers and was calculated with the formula V = (L×W^2^)×0.5. Photographs of dissected tumors from nude mice (B) and the curve of tumor growth (C) are shown. *:P<0.05. Results are representative of three animals in every time point per group. D:The level of VCP and Bcl-2 in the transplanted tumors was detected by IHC.

Although it has been reported that VCP expression level is diverse in the different tumor samples and has prognostic significance for disease-free and overall survival of patients with HCC [2], the role of VCP in HCC is unclear. So we wondered whether VCP could influence the progression of HCC or just be a potential prognostic marker. Firstly, qRT-PCR experiment was performed to analyze the level of VCP in 11 paired of HCC frozen tissues and its adjacent normal frozen tissues. It was showed that the level of VCP was significantly increased in 9 HCC tissues (Ratio>2, P<0.05) compared with the corresponding normal liver tissues (Figure 1A). In the further investigation, the expression of VCP was knocked down in HepG2 cells with specific siRNA (si-VCP), and then the cells were injected subcutaneously into the posterior flank of the male BALB/c athymic nude mouse. Compared with NC transfectants, cells transfected with si-VCP revealed a delayed tumor formation time (11/15 versus 0/15 on day 10) and a significant reduction in the tumor size (Figure 1B, 1C). In addition, the immunohistochemical analysis showed that the level of VCP in the tumor from si-VCP group was lower than that from NC group (Figure 1D). At the same time, we tested the level of Bcl-2 in the different groups. Bcl-2 was an anti-apoptotic protein, which was an important regulator of programmed cell death and apoptosis [23], [24]. It was found that the expression of Bcl-2 was decreased in the si-VCP group (Figure 1D). These results suggested that down-regulation of VCP expression could obviously suppress tumor growth *in vivo*.

### miR-129-5p could directly regulate the expression of VCP

**Figure 2:**
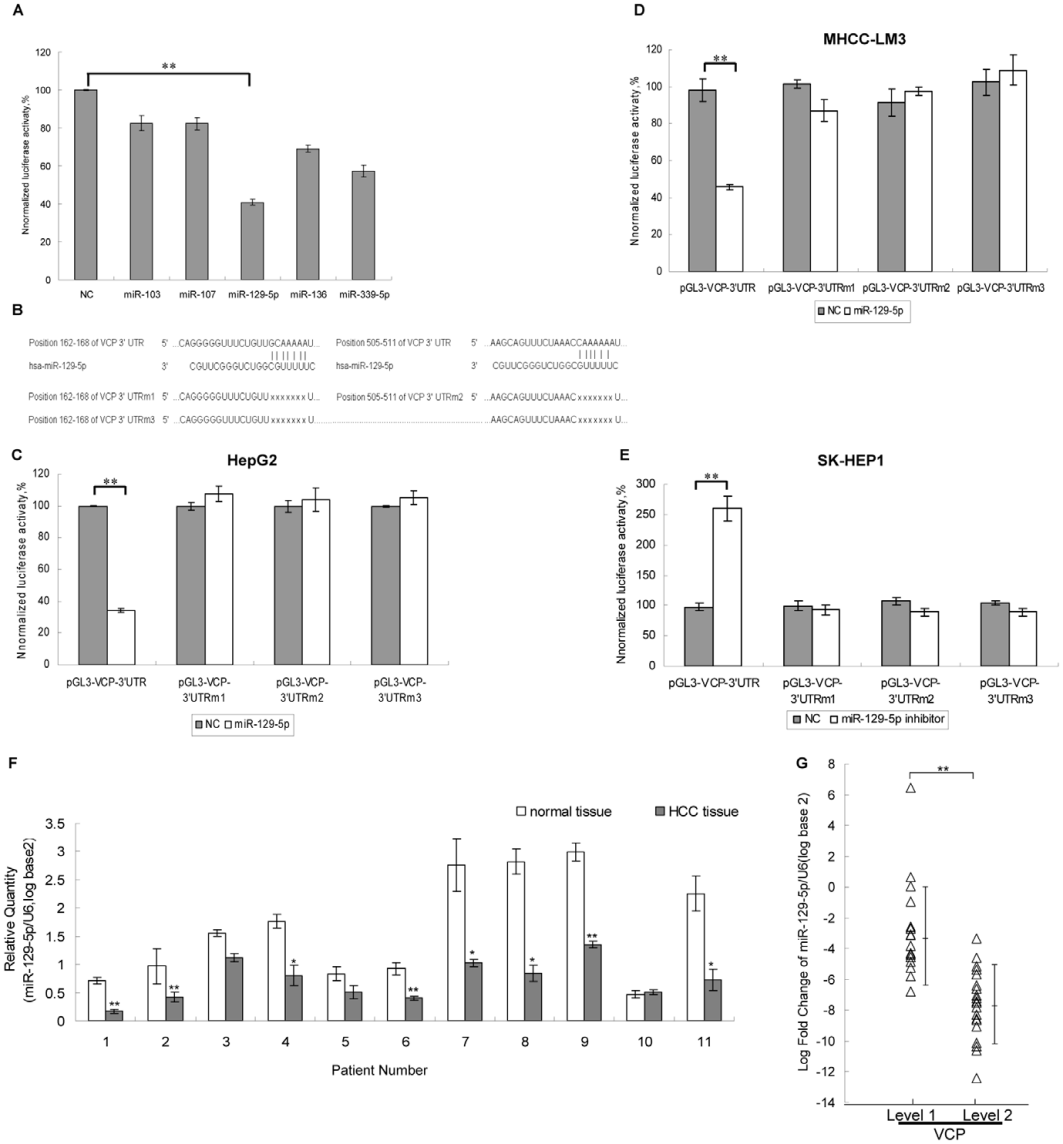
miR-129-5p could directly regulate the expression of VCP. A: miR-129-5p could significantly suppress the luciferase activities of pGL3-VCP-3′UTR in HepG2 cells. Five predicted miRNAs (miR-103, miR-107, miR-129-5p, miR-136, miR-339-5p) were transfected in HepG2 cells, respectively, with VCP 3′UTR report. 72 h after the transfection, the cells were harvested for luciferase activity. Shown data are representative from three independent experiments. **:P<0.01. B: Bioinformatic analysis of miR-129-5p predicted binding sites in the VCP 3′UTR. There were two putative miR-129-5p target sites located in the VCP 3′UTR (162–168 and 505–511). Three mutant reporter vectors were constructed with the deletion of two target sites individually (pGL3-VCP-3′UTRm1, pGL3-VCP-3′UTRm2) or both (pGL3-VCP-3′UTRm3). C: The luciferase activity of the mutant VCP 3′UTR report genes were not regulated by miR-129-5p in HepG2 cells. Shown data are representative from three independent experiments.**:P<0.01. D: miR-129-5p could significantly suppress the luciferase activities of pGL3-VCP-3′UTR in MHCC-LM3 cells, while has no effect on three mutant reporter vectors. Shown data are representative from three independent experiments.**:P<0.01. E: The inhibiter of miR-129-5p could increase the luciferase activities of pGL3-VCP-3′UTR in SK-HEP1 cells, while has no effect on three mutant reporter vectors. Shown data are representative from three independent experiments. **:P<0.01. F: Analysis of the level of miR-129-5p in HCC tissues by qRT-PCR. The result showed that the level of miR-129-5p is frequently reduced in human HCC tissues.*:P<0.05.**:P<0.01. G: The level of miR-129-5p was negatively correlated with the expression of VCP in HCC. The 39 tissue samples of HCC were divided into two groups according to the level of VCP. The level of miR-129-5p in each sample from the two groups (level 1,n = 17;level 2,n = 22) was measured by qRT-PCR and compared. U6 snRNA was used as internal control gene. Dot plots showed an inverse relationship between U6 snRNA and miR-129-5p expression in HCC. Significant differences were determined using Student's t tests. **: P<0.01; Bars: mean±SD.

The level of VCP is altered in HCC tissue samples^2^ and important for tumor growth. So we tried to investigate the molecule which could regulate the expression of VCP in HCC. MiRNAs have been reported to regulate the expression of various proteins and involved in many kinds of cancer. So we investigated if there were some miRNAs which could regulate the expression of VCP in HCC. We firstly predicted the candidates by using the Targetscan database (www.targetscan.org). Five potential candidate miRNAs (miR-103; miR-107; miR-129-5p; miR-136; miR-339-5p) were predicted to have target sites in 3′UTR of VCP mRNA. To test which miRNA can regulate the expression of VCP, a reporter vector (pGL3-VCP-3′UTR) was created by insertion of the 3′UTR of VCP mRNA to the downstream of the luciferase gene. The reporter vector was transfected into HepG2 cells together with miRNAs or negative control RNA duplex (NC). 48 hours after transfection, the luciferase activities were tested. It was found that only miR-129-5p can down-regulate the luciferase activities of the reporter (Figure 2A). miR-129-5p is produced by the precursor of *miR-129-1* and *miR-129-2* and both of them negatively regulate SOX4 [25]. There were two predicted miR-129-5p target sites in the 3′UTR of VCP mRNA, 162–168 and 505–511. To confirm the binding between miR-129-5p and 3′UTR of VCP, three mutants of 3′UTR of VCP mRNA were constructed by deleting the two targets sites individually or both to generate three reporter vectors(pGL3-VCP-3′UTRm1/m2/m3)(Figure 2B). The three mutant reporters were transfected into two HCC cell lines (HepG2 and MHCC-LM3) together with miR-129-5p. The luciferase expression was no longer regulated by miR-129-5p after the 162–168 and/or 505–511 of 3′UTR were deleted (Figure 2C, 2D). This suggested that both target sites in the 3′UTR of VCP mRNA were essential for the regulation of miR-129-5p.

To further verify the regulatory role of miR-129-5p on VCP expression, the inhibitor of miR-129-5p was transfected into the liver cancer cell line SK-HEP1 together with pGL3-VCP-3′UTR. The level of miR-129-5p was higher in SK-HEP1 than that in HepG2 and MHCC-LM3 (data not shown). It was found that the luciferase activities in SK-HEP1 cells were increased after the cells were transfected with miR-129-5p inhibitor (Figure 2E). In addition, no significant difference in the luciferase activities of pGL3-VCP-3′UTRm1/m2/m3 was observed after the cells were transfected with the inhibitor of miR-129-5p (Figure 2C,2D,2E). These results suggested that miR-129-5p directly interacts with the 3′UTR of VCP mRNA.

In the further investigation, we analyzed the level of miR-129-5p in 11 paired HCC and the corresponding normal liver tissues by qRT-PCR. The significant decreased level of miR-129-5p was observed in HCC tissues (Figure 2F).

To verify the correlation between VCP and miR-129-5p, the level of VCP in the paraffin-embedded tissue samples of HCC was detected by immunohistochemistry with specific antibodies against VCP. Then these samples were divided into two groups (level 1(n = 17) and level 2 (n = 22)) according the level of VCP as the classification standard described previously [2] (Table S2) in which the expression of VCP in level 1 was lower than that in level 2. At the same time, the expression level of miR-129-5p in two VCP level groups was measured by qRT-PCR. It was found that the level of miR-219-5p was higher in level 1 than that in level 2, which indicated the miR-129-5p level was negatively related to the expression of VCP (Figure 2G). It was found that miR-129-5p could also suppress the progression of HCC *in vivo*. Cells transfected with miR-129-5p revealed a delayed tumor formation time (11/15 versus 2/15 on day 10) and a significant reduction in the tumor size, which was consist with the result of si-VCP group, suggesting a potential tumor suppressive effect of miR-129-5p (Figure 1B, 1C).

### miR-129-5p suppressed the expression of VCP protein and regulated NF-κB pathway

**Figure 3:**
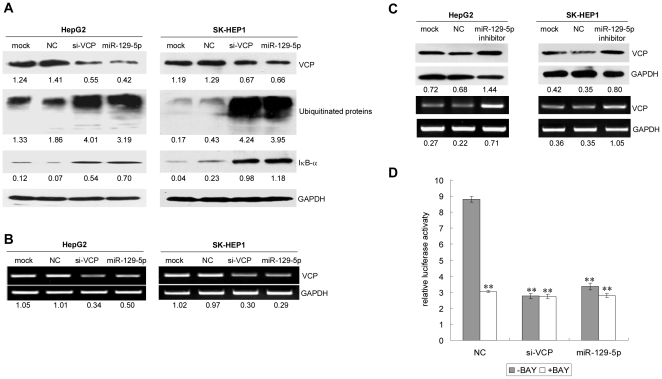
miR-129-5p could decrease the level of VCP and increase the level of accumulated unbiqutinated proteins and IκBα. A: Detection the level of VCP protein, accumulated ubiquitinated proteins and IκBα by Western blot after miR-129-5p, siRNA of VCP and NC were transfected into HepG2 or SK-HEP1 cells individually for 72 h. GAPDH served as the internal control. The experiments were repeated three times independently. The value under each lane indicates the relative expression level of protein, which was represented by the intensity ratio between VCP/Ubiquitinated proteins/IκBα and GAPDH fragments in each lane. B: Detection of the level of VCP mRNA in HepG2 or SK-HEP1 cells after transient transfection with si-VCP or miR-129-5p for 72 h. GAPDH served as the internal control. The experiment was repeated three times independently. The value under each lane indicates the relative level of VCP mRNA, which was represented by the intensity ratio between VCP and GAPDH fragments in each lane. C: Detection the level of VCP protein by Western blot (Upper panel) and mRNA by RT-PCR (Lower panel) after miR-129-5p inhibitor and NC were transfected into HepG2 or SK-HEP1 cells individually for 72 h. GAPDH served as the internal control. The experiments were repeated three times independently. The value under each lane indicates the relative level of VCP protein (Upper panel) or mRNA (Lower panel), which was represented by the intensity ratio between VCP and GAPDH fragments in each lane. D: Detection of the activities of NF-κB. The reporter vector of NF-κB was transfected into HepG2 together with miR-129-5p. At the same time, the NF-κB inhibitor (BAY 11-7082) was added. The activities of luciferase were detected. Shown data are representative from three independent experiments.**:P<0.01.

**Figure 4:**
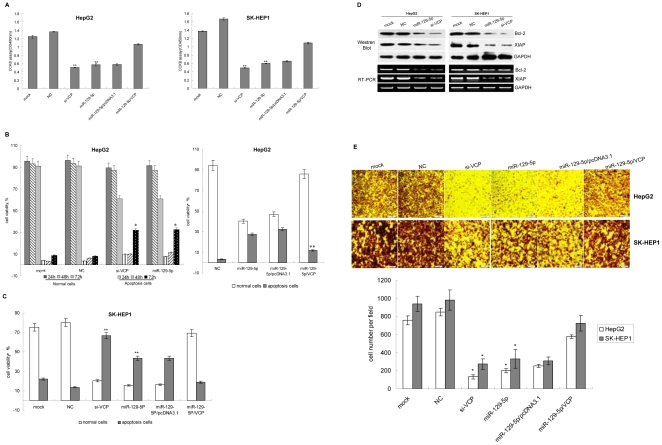
miR-129-5p could regulate the cell growth, apopotosis and migration of HCC cells dependent on the regulation of VCP expression. A: HepG2 or SK-HEP1 cells were transfected and in the indicated time periods posttransfection, cell growth rate was evaluated using CCK8 assay. The result showed miR-129-5p and si-VCP could repress cell growth. The cell growth rate was restored after VCP expression vector were transfected into HepG2 or SK-HEP1 cells. Shown data are representative from three independent experiments. **:P<0.01. B: HepG2 cells were transfected and the apoptosis of cell was detected with FACS. Left panel showed miR-129-5p and si-VCP could increase the apoptosis of HepG2 cells at 48 h and 72 h after the transfection. Right panel showed the apoptosis of cell after miR-129-5p with/without VCP expression vector were transfected into HepG2 cells for 72 h. The experiment was repeated three times independently. NC: negative control RNA duplex; miR-129-5p: miR-129-5p mimic; miR-129-5p/pcDNA3.1: cells were co-transfected with miR-129-5p mimic and blank pcDNA3.1; miR-129-5p/VCP: cells were co-transfected with miR-129-5p mimic and VCP expression vector. The experiments were repeated three times independently.*:P<0.05. **:P<0.01. C: SK-HEP1 cells were transfected and the apoptosis of cells was detected with FACS. miR-129-5p and si-VCP could increase the apoptosis of SK-HEP1 cells at 72 h after the transfection. After the level of VCP was enhanced by transfecting VCP expression vector, the apoptosis rate of cells were returned. D:Detection the level of Bcl-2 and XIAP by RT-PCR and western blot. GAPDH served as the internal control. E:Transwell assay was performed to assess cell migration. Upper panel represented the photographs of treated and untreated cells at 24 h (×40 magnification). Lower panel showed the number of cells invaded at 24 h. Shown data are representative from four replicates per group. *P<0.05.

To verify whether VCP protein expression was indeed regulated by miR-129-5p, miR-129-5p, si-VCP or NC were transfected into HepG2 and SK-HEP1 cells. The western blot showed that the expression of VCP protein was significant reduced after the cell was treated with miR-129-5p or si-VCP compared with NC (Figure 3A). The reduction was also observed in the mRNA level of VCP in the cells transfected with si-VCP or miR-129-5p (Figure 3B). In the further investigation, it was found that the expression of VCP protein was enhanced compared to NC group after the miR-129-5p inhibitor was transfected to cells (Figure 3C). It has been reported that VCP protein is involved in the degradation of ubiquitinated proteins. Figure 4A showed that the ubiquitinated proteins were accumulated after the cells were treated with miR-129-5p or si-VCP. In addition, VCP could interact with the ubiquitinated IκBα, the inactivation of the prototype NF-κB inhibitor, and play an important role in the proteasome-mediated degradation of IκBα [7]. In our experiment, we found the level of IκBα in the HepG2 and SK-HEP1 cells after transfection with miR129-5p or si-VCP was increased (Figure 3A). These results indicated miR-129-5p might modulate the NF-κB signal pathway through the regulation of VCP.

In the further investigation, we checked if miR-129-5p or si-VCP could regulate the NF-κB signal pathway. To assess whether the activities of NF-κB was affected by VCP or miR-129-5p, we transiently transfected a reporter plasmid with a luciferase gene linked to the binding region of NF-κB (pNFκB-luc) in HepG2 cells. Cells were harvested at 72 h after transfection. As shown in Figure 3D, the luciferase activities of pNFκB-luc were strongly repressed following miR-129-5p or si-VCP transfection. When cells had been treated with the NF-κB inhibitor (BAY 11-7082), miR-129-5p or si-VCP could not influence the activities of NF-κB. These results suggested that miR-129-5p and VCP was involved in the NF-κB signal pathway through degradation of IκBα.

### miR-129-5p induced the apoptosis and reduced the migration of HCC cells dependent on the down-regulation of VCP expression

Resistance towards apoptosis is a key factor for the survival of a malignant cell and cancer cell migration to distant organs is the major cause of death in almost all forms of cancer [26], [27]. Moreover, the NF-κB signal pathway is involved in tumor growth and metastasis. So we checked if miR-129-5p or VCP could influence the cell growth, apoptosis and migration of HCC cells. HepG2 and SK-HEP1 cells were transfected with miR-129-5p or si-VCP. Then the cell growth was evaluated using CCK8 assay and the apoptosis was detected by flow cytometry. It was found that silencing of VCP gene with si-VCP suppressed cell growth (Figure 4A) and increased the apoptosis of both HepG2 and SK-HEP1 cells, which was consistent with the consequence of restoration of miR-129-5p expression (Figure 4B, 4C). In addition, we detected the expression of Bcl-2 and XIAP which were the NF-κB-regulated target genes and involved in the different pathway of cell apoptosis. It was found that the level of Bcl-2 and XIAP was decreased in HepG2 or SK-HEP1 cells transfected with miR-129-5p or si-VCP (Figure 4D). We also measured the migration of the HepG2 or SK-HEP1 cells by transwell chemotaxis assay. It was found that the number of cells migrating across the membranes was decreased dramatically after the cells were treated with miR-129-5p or si-VCP. The migration level of miR-129-5p group was reduced to 23.54% of negative control level. Similarly, the level of si-VCP group was reduced to 15.56% (Figure 4E).

To further confirm the effect of miR-129-5p was dependent on the regulation of VCP expression, the VCP was expressed in HCC cells transfected with miR-129-5p. And then the cell growth, apoptosis and migration of cells were analyzed respectively. The results showed that after the VCP level was recovered, the cell growth and migration were increased and the apoptosis rate was reduced (Figure 4A, 4B, 4C, 4E). It suggested that phenotypic alternation of cells induced by miR-129-5p was achieved by direct influencing the expression of VCP.

## Discussion

Yamamoto S *et al* identified that HCC patients with VCP-level 2 showed higher rate of portal vein invasion in the tumor and poorer disease-free and overall survival compared with level 1 patients [2]. It has been also reported that the level of VCP is associated with the prognosis of other kinds of carcinoma, including prostate cancer, esophageal carcinoma, gingival squamous cell carcinoma and colorectal carcinomas [28]–[32]. All these findings indicate that VCP can be used as a potential marker of tumor. Until now, there are no definitive reports to clarify if VCP is involved in the progression of tumor. Here, we demonstrated the elevated level of VCP in HCC tissues. Inhibition of VCP could suppress HCC tumor progression in nude mice. The size of tumors from si-VCP group was significantly lower than that from NC group.

Up to now, the regulatory mechanism of VCP expression is rarely known. In this study, we identified that miR-129-5p could down-regulate the expression of VCP by interaction with two sites located at its 3′UTR. Further investigation revealed that miR-129-5p could inhibit the degradation of IκBα. IκBα is the inhibitor of NF-κB, so the affection on the cell growth, apoptosis and migration induced by VCP and miR-129-5p might be via NF-κB pathway. Given the broad association between VCP and various cell activities, further study on whether miR-129-5p is involved in these processes will conducted in the future.

The microarray results in the previous reports had presented that the level of miR-129 was deregulated in human HCC tissues compared with the normal controls [16]. In our study, it was found that miR-129-5p was frequently decreased in HCC, which was in accordance with the previous reports [17]. In the HCC tissues, it was found that the expression of miR-129-5p was negatively correlated with the level of VCP. The *in vivo* study showed that enhancing the level of miR-129-5p could suppress the growth of tumor, which was similar to si-VCP group. All of these results revealed miR-129-5p may be associated with the progression of HCC by inhibiting the expression of VCP.

As described previously, miR-129-5p origins from *miR-129-1* and *miR-129-2*. Huang Y *et al* identified that the miR-129-2 CpG island was frequently hypermethylated in endometrial tumors and the level of miR-129-5p decreases [33]. Wu et al reported that miR-129-5p could regulate the expression of Cdk6 and induce G1 phase arrest in mouse lung epithelial cell line (E10 Cells) [34]. But this phenomenon was observed in our experiment (data not shown). This may be due to the different cell lines used. It will be investigated whether miR-129-2 is hypermethylated in HCC and Cdk6 is regulated by miR-129-5p in HCC cell lines in the future.

In conclusion, our results identified that miR-129-5p can directly inhibit the expression of VCP. This regulation plays an important role in cell apoptosis *in vitro* and the development of HCC *in vivo*. As the level of VCP and miR-129-5p in HCC samples is significantly different from that in adjacent normal tissues, the two molecules may be an indicator for prediction of the genesis of HCC. Our study may lead to the development of new therapeutic regimens in treating advanced HCC.

## Supporting Information

Table S1 **The patient clinical feature of HCC and control tissue specimens.**
(DOC)

Table S2 **The patient clinical feature of paraffin-embedded tissues specimens.**
(DOC)

Table S3 **Primers.**
(DOC)
