# Supplementary material for: VCP/p97, Down-Regulated by microRNA-129-5p, Could Regulate the Progression of Hepatocellular Carcinoma
Source: PLoS One. 2012 Apr 20;7(4):e35800. doi: 10.1371/journal.pone.0035800 (PMC3335000; doi:10.1371/journal.pone.0035800)
Supplement: Table S1 — The patient clinical feature of HCC and control tissue specimens. (DOC) [file pone.0035800.s001.doc]

**Table S**1

| Patient No. | 1 | 2 | 3 | 4 | 5 | 6 | 7 | 8 | 9 | 10 | 11 |
| --- | --- | --- | --- | --- | --- | --- | --- | --- | --- | --- | --- |
| Sex | F | M | F | M | F | M | M | M | F | M | M |
| Age | 41 | 34 | 51 | 33 | 35 | 63 | 70 | 74 | 69 | 42 | 54 |
| Tumer Size (cmХcmХcm） | 12x10x8 | 10x7 | 9x10 | 20x18 | 5x4 | 14x10x10 | 3.5x3.5x3.5 | 9x9x9 | 15x7x5 | 12x11x16 | 2.5x2x2 |
| HbsAg(P/N) | P | P | P | P | P | P | P | N | P | P | P |
| HCV-b(P/N) | N | N | N | N | N | N | N | N | N | N | N |
| cirrhosis | No | Yes | Yes | Yes | Yes | Yes | Yes | Yes | No | No | No |
| AFP(>/<200ng/ML) | < | < | > | > | < | < | < | > | < | < | < |
| TNM stage | III | II | II | III | II | III | II | III | III | III | II |
| Edmondson  Grade | II | III | II | III | II | II | III | II | III | III | II |
