# Supplementary material for: VCP/p97, Down-Regulated by microRNA-129-5p, Could Regulate the Progression of Hepatocellular Carcinoma
Source: PLoS One. 2012 Apr 20;7(4):e35800. doi: 10.1371/journal.pone.0035800 (PMC3335000; doi:10.1371/journal.pone.0035800)
Supplement: Table S2 — The patient clinical feature of paraffin-embedded tissues specimens. (DOC) [file pone.0035800.s002.doc]

**Table S2**

| Patient No. | 1 | 2 | 3 | 4 | 5 | 6 | 7 | 8 | 9 | 10 | 11 | 12 | 13 |
| --- | --- | --- | --- | --- | --- | --- | --- | --- | --- | --- | --- | --- | --- |
| Sex | M | M | M | M | M | M | M | M | M | M | M | M | M |
| Age | 44 | 50 | 53 | 43 | 51 | 41 | 52 | 44 | 44 | 49 | 53 | 64 | 50 |
| Tumer Size (cmХcmХcm） | 2.5x2x2 | 17x11.5x9 | 4.5x3.5x13 | 3x2.5x2.5 | 6x4x4 | 2.5x2x2 | 2x2x1.5 | 2.5x2x2 | 3x2.5x2.5 | 3x2.5x2.5 | 7x6x5 | 4.5x4.5x4.5 | 4.2x4x2.9 |
| HbsAg(P/N) | P | N | P | P | N | P | P | P | P | P | P | N | P |
| HCV-b(P/N) | N | N | N | N | N | N | N | N | N | N | N | P | N |
| cirrhosis | Yes | Yes | Yes | Yes | No | No | Yes | Yes | No | No | Yes | Yes | Yes |
| AFP(>/<200ng/ML) | < | < | < | < | < | < | < | < | < | < | > | > | < |
| TNM stage | II | III | III | II | III | II | I | II | II | II | III | III | III |
| Edmondson  Grade | II | III | II | II | III | III | II | II | III | III | II | II | II |
| VCP LEVEL***** | 1 | 1 | 1 | 1 | 1 | 1 | 1 | 1 | 2 | 2 | 2 | 2 | 2 |
|  | | | | | | | | | | | | | |
| Patient No. | 14 | 15 | 16 | 17 | 18 | 19 | 20 | 21 | 22 | 23 | 24 | 25 | 26 |
| Sex | M | M | M | M | F | M | M | M | M | M | M | F | M |
| Age | 56 | 55 | 48 | 59 | 55 | 55 | 47 | 56 | 59 | 59 | 49 | 57 | 63 |
| Tumer Size (cmХcmХcm） | 3.8x3.8x3.5 | 4.5x4.5x4.5 | 2x2x1.8 | 3x1x1 | 2x1.5x1.5 | 2.5x1.5x1 | 4x2x1 | 7x 1x0.5 | 6x5x4 | 12x10x7 | 4.5x4.2x2.8 | 2.5x2.5x2 | 1x1x0.8 |
| HbsAg(P/N) | N | P | P | P | N | P | P | P | N | P | P | N | P |
| HCV-b(P/N) | N | N | N | N | N | N | N | N | N | N | N | P | N |
| cirrhosis | Yes | No | No | Yes | Yes | Yes | Yes | Yes | Yes | Yes | Yes | Yes | Yes |
| AFP(>/<200ng/ML) | > | > | < | < | < | > | < | > | < | < | < | < | < |
| TNM stage | II | III | I | II | I | II | II | III | III | III | II | II | I |
| Edmondson  Grade | II | III | II | III | II | II | III | II | III | III | II | III | II |
| VCP LEVEL ***** | 2 | 2 | 2 | 1 | 1 | 1 | 1 | 1 | 1 | 1 | 1 | 1 | 2 |
|  | | | | | | | | | | | | | |
| Patient No. | 27 | 28 | 29 | 30 | 31 | 32 | 33 | 34 | 35 | 36 | 37 | 38 | 39 |
| Sex | M | M | M | M | M | M | M | F | M | M | M | M | M |
| Age | 40 | 50 | 56 | 52 | 58 | 46 | 47 | 50 | 69 | 43 | 53 | 45 | 58 |
| Tumer Size (cmХcmХcm） | 7.5 x1x0.5 | 5x4x3 | 2x2x1.5 | 3.5x0.5x0.5 | 10.5x10x7 | 16x11x8 | 2.5x2x1 | 2.5x1.5x1.0 | 3.5x3x3 | 3x3x2 | 1.5x1.0x1.0 | 6x5x5 | 2x2x1 |
| HbsAg(P/N) | P | P | P | P | P | P | P | P | P | P | P | P | P |
| HCV-b(P/N) | N | N | N | N | N | N | N | N | N | N | N | P | N |
| cirrhosis | Yes | Yes | No | Yes | Yes | Yes | Yes | Yes | Yes | Yes | Yes | Yes | Yes |
| AFP(>/<200ng/ML) | < | < | < | > | > | > | > | < | > | > | > | < | > |
| TNM stage | III | III | II | II | III | III | II | II | II | III | I | III | II |
| Edmondson  Grade | II | II | III | III | II | III | III | II | III | II | II | III | III |
| VCP LEVEL ***** | 2 | 2 | 2 | 2 | 2 | 2 | 2 | 2 | 2 | 2 | 2 | 2 | 2 |

*****:VCP LEVEL: tissue samples of HCC were divided into two groups according the level of VCP which was detected by immunohistochemistry.
