# Supplementary material for: VCP/p97, Down-Regulated by microRNA-129-5p, Could Regulate the Progression of Hepatocellular Carcinoma
Source: PLoS One. 2012 Apr 20;7(4):e35800. doi: 10.1371/journal.pone.0035800 (PMC3335000; doi:10.1371/journal.pone.0035800)
Supplement: Table S3 — Primers. (DOC) [file pone.0035800.s003.doc]

**Table S3**

| Primers | Sequence |
| --- | --- |
| miR129 RTprimer | GTCGTATCCAGTGCAGGGTCCGAGGTATTCGCACTGGATACGAC GCAAGC |
| miR-103 RTprimer | GTCGTATCCAGTGCAGGGTCCGAGGTATTCGCACTGGATACGAC TCATAG |
| miR-107 RTprimer | GTCGTATCCAGTGCAGGGTCCGAGGTATTCGCACTGGATACGAC TGATAG |
| miR-339 RTprimer | GTCGTATCCAGTGCAGGGTCCGAGGTATTCGCACTGGATACGAC CGTGAG |
| miR-136 RTprimer | GTCGTATCCAGTGCAGGGTCCGAGGTATTCGCACTGGATACGAC TCCATC |
| miRNA RTprimer | GTGCAGGGTCCGAGGT |
| miR -129 RT5’P | cTTTTTGCGGTcTGGGCTTGC |
| miR -103 RT5’P | AGCAGCATTGTACAGGGCTATGA |
| miR -107 RT5’P | AGCAGCATTGTACAGGGCTATCA |
| miR -339 RT5’P | TCCCTGTCCTCCAGGAGCTCACG |
| miR -136 RT5’P | ACTCCATTTGTTTTGATGATGGA |
| VCP RT5’P | CCCTGTGCCTGCTTCTTT |
| VCP RT3’P | GCTGCTCCCTTTCCACCA |
| U6 RT5’P | TAAAATCTATATACACGACGGCTTCG |
| U6 RT3’P | TACTGTGCGTTTAAGCACTTCGC |
| GAPDH RT5’P | AGAAGGCTGGGGCTCATTTG |
| GAPDH RT3’P | AGGGGCCATCCACAGTCTTC |
